# Quantitative background parenchymal uptake on molecular breast imaging and breast cancer risk: a case-control study

**DOI:** 10.1186/s13058-018-0973-3

**Published:** 2018-06-05

**Authors:** Carrie B. Hruska, Jennifer R. Geske, Tiffinee N. Swanson, Alyssa N. Mammel, David S. Lake, Armando Manduca, Amy Lynn Conners, Dana H. Whaley, Christopher G. Scott, Rickey E. Carter, Deborah J. Rhodes, Michael K. O’Connor, Celine M. Vachon

**Affiliations:** 1 Department of Physiology and Biomedical Engineering, Mayo Clinic, 200 First Street SW, Rochester, MN 55905 USA; 2 Department of Health Sciences Research, Mayo Clinic, 200 First Street SW, Rochester, MN 55905 USA; 3 Department Medicine, Mayo Clinic, 200 First Street SW, Rochester, MN 55905 USA

**Keywords:** Breast density, Breast cancer risk, Molecular breast imaging, Tc-99m sestamibi, Mammography

## Abstract

**Background:**

Background parenchymal uptake (BPU), which refers to the level of Tc-99m sestamibi uptake within normal fibroglandular tissue on molecular breast imaging (MBI), has been identified as a breast cancer risk factor, independent of mammographic density. Prior analyses have used subjective categories to describe BPU. We evaluate a new quantitative method for assessing BPU by testing its reproducibility, comparing quantitative results with previously established subjective BPU categories, and determining the association of quantitative BPU with breast cancer risk.

**Methods:**

Two nonradiologist operators independently performed region-of-interest analysis on MBI images viewed in conjunction with corresponding digital mammograms. Quantitative BPU was defined as a unitless ratio of the average pixel intensity (counts/pixel) within the fibroglandular tissue versus the average pixel intensity in fat. Operator agreement and the correlation of quantitative BPU measures with subjective BPU categories assessed by expert radiologists were determined. Percent density on mammograms was estimated using Cumulus. The association of quantitative BPU with breast cancer (per one unit BPU) was examined within an established case-control study of 62 incident breast cancer cases and 177 matched controls.

**Results:**

Quantitative BPU ranged from 0.4 to 3.2 across all subjects and was on average higher in cases compared to controls (1.4 versus 1.2, *p* < 0.007 for both operators). Quantitative BPU was strongly correlated with subjective BPU categories (Spearman’s *r* = 0.59 to 0.69, *p* < 0.0001, for each paired combination of two operators and two radiologists). Interoperator and intraoperator agreement in the quantitative BPU measure, assessed by intraclass correlation, was 0.92 and 0.98, respectively. Quantitative BPU measures showed either no correlation or weak negative correlation with mammographic percent density. In a model adjusted for body mass index and percent density, higher quantitative BPU was associated with increased risk of breast cancer for both operators (OR = 4.0, 95% confidence interval (CI) 1.6–10.1, and 2.4, 95% CI 1.2–4.7).

**Conclusion:**

Quantitative measurement of BPU, defined as the ratio of average counts in fibroglandular tissue relative to that in fat, can be reliably performed by nonradiologist operators with a simple region-of-interest analysis tool. Similar to results obtained with subjective BPU categories, quantitative BPU is a functional imaging biomarker of breast cancer risk, independent of mammographic density and hormonal factors.

**Electronic supplementary material:**

The online version of this article (10.1186/s13058-018-0973-3) contains supplementary material, which is available to authorized users.

## Background

Mammographic density, or the amount of fibroglandular tissue in the breast as depicted on a mammogram, is known to reduce the accuracy of mammography in detecting cancer [1–3]. Density is also independently associated with breast cancer risk as established by numerous analyses conducted over the last 40 years, consistently showing women with the densest breasts to be four- to six-times more likely to be diagnosed with breast cancer compared with those with low density [4, 5]. However, because breast density is highly prevalent (approximately 40 to 50% of screening-eligible women have heterogeneously or extremely dense breasts according to American College of Radiology (ACR) Breast Imaging-Reporting and Data System (BI-RADS) categories [6, 7]), it is impractical for clinicians to consider all women with dense breasts to be at elevated risk since doing so would warrant consideration of supplemental screening or preventive options in nearly half the screening population. To identify the subset of women with dense breasts at greatest risk of breast cancer, and those most likely to benefit from these strategies, improved risk stratification tools are needed.

**Fig. 1:**
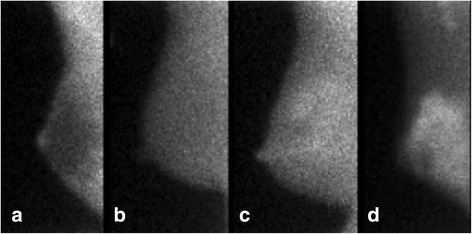
BPU subjective categories. Example MBI images from four women, all acquired in right mediolateral oblique projection, showing the range of BPU observed: **a** photopenic BPU, **b** minimal to mild BPU, **c** moderate BPU, and **d** marked BPU

Molecular breast imaging (MBI) is a nuclear medicine test that uses dedicated gamma cameras and injection of a radiotracer, typically Tc-99m sestamibi, to detect breast cancer. As MBI is a functional imaging technique that relies on the preferential uptake of radiotracer in metabolically active cells, it is able to reveal cancers obscured by breast density on mammography. MBI can also depict the functional uptake of radiotracer in benign fibroglandular tissue, which has been termed background parenchymal uptake (BPU). High levels of BPU are hypothesized to represent breast tissue with elevated metabolic activity due to a combination of factors such as abundant mitochondria, cellular proliferation, and blood flow [8, 9]. Among women with similar mammographic density, BPU has been observed to vary substantially from a lack of uptake in fibroglandular tissue (photopenic) to very high intensity uptake (marked), as shown in Fig. 1. Importantly, subjective categories of high BPU were found to be associated with risk of incident breast cancer relative to those with low BPU in a case-control analysis (odds ratio (OR) range from 3 to 5) after adjustment for mammographic density and exogenous hormone use [10]. These results suggest that BPU is a functional imaging biomarker that depicts risk-related aspects of fibroglandular tissue not observed through measures of mammographic density alone.

Prior investigations of BPU on MBI have used a subjective measure which includes the following four categories: photopenic, minimal to mild, moderate, and marked [11, 12]. While expert readers were observed to have substantial agreement using this subjective classification (κ = 0.84) [13], only fair agreement was observed among nonexpert readers (κ = 0.31) [12]. A quantitative tool for BPU assessment may improve reproducibility of the measure. Additionally, a quantitative measure of BPU on a continuous scale would provide a more precise measurement and could therefore more accurately monitor changes in BPU over serial MBI examinations.

The objective of this work was to develop and evaluate a new quantitative method for assessing BPU by assessing its reproducibility, comparing quantitative to categorical BPU measures, and determining the association of quantitative BPU with breast cancer risk.

## Methods

### Study population

This retrospective analysis was compliant with the US Health Insurance Portability and Accountability Act and approved by the Mayo Clinic Institutional Review Board, which issued a waiver of informed consent. A case-control study previously established to evaluate the association of subjective BPU categories with breast cancer risk was used in the current analysis [10]. As previously described, we established this case-control group within a cohort of patients who underwent MBI between 1 February 2005 and 28 February 2014 (*n* = 3202) and who were followed for breast cancer diagnoses through 31 December 2014. A total of 3027 patients were eligible for study inclusion as they had provided general authorization to use medical records in research, did not have breast implants at the time of MBI, and did not have a prior history of breast cancer or were diagnosed at the time of MBI (within 180 days).

Any subject with a diagnosis of invasive breast cancer or ductal carcinoma in situ (DCIS) 180 days or more following the MBI was considered a case. Sixty-two cases were identified; the median time from MBI to diagnosis was 3.1 years. Control subjects were matched to cases on age (within 5 years), menopausal status, year of MBI, and follow-up interval (at least as long as matched case); the median follow-up time was 6.1 years. Up to three controls per case were originally selected (*n* = 179). Two controls were excluded as their mammograms were unavailable for use in the quantitative BPU software program (described below) leaving 177 controls for the current analysis.

Patient information at the time of MBI, including body mass index (BMI), menopausal status, postmenopausal hormone use, breast biopsy history, and family history of breast cancer, was obtained from research study questionnaires and medical record review.

### Images

**Fig. 2:**
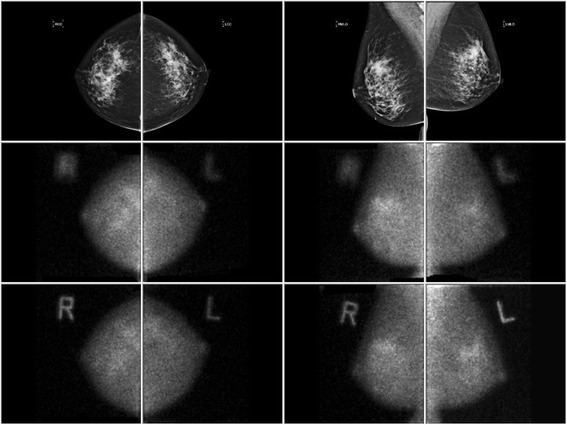
Example layout of images for quantitative BPU region-of-interest analysis. Bilateral mammogram and MBI views in CC and MLO projections are displayed. Mammogram in top row (from left to right) comprises right CC, left CC, right MLO, and left MLO projections. The same projections acquired by MBI are shown for the upper detector (middle row) and for the lower detector (bottom row)

MBI examinations were performed as previously described [10]. Briefly, MBI was performed using a dedicated dual-head gamma camera system equipped with semiconductor-based detectors (cadmium zinc telluride). Following injection of Tc-99m sestamibi, two-view acquisitions (craniocaudal (CC) and mediolateral oblique (MLO)) of each breast were made. Thus, the entire MBI dataset comprised of eight images: a CC and MLO projection of left and right breasts acquired with two detector heads of the dual head system (Fig. 2).

Mammograms performed closest to the time of MBI were used for density analysis. The median time from mammogram to MBI was 0 days (interquartile range (IQR) 0–1 days). Mammographic density was classified according to the ACR BI-RADS breast composition categories (4th edition) at the time of clinical interpretation [14]. Density was also quantitatively measured as percent density (PD) by a trained operator using a semi-automated software tool (Cumulus; University of Toronto, Toronto, ON, Canada [15]), as previously described [16].

### Quantitative BPU measurement

The subjective BPU categories, as defined in a validated lexicon for gamma imaging of the breast [11, 12], are intended to describe the relative intensity of radiotracer uptake in normal breast parenchyma (or fibroglandular tissue) compared with intensity of uptake in subcutaneous fat. These categories and their definitions are as follows: 1) photopenic BPU, fibroglandular intensity less than fat intensity; 2) minimal to mild BPU, fibroglandular intensity equal to or slightly greater than fat intensity; 3) moderate BPU, fibroglandular intensity greater than mild but less than twice as intense as fat; and 4) marked BPU, fibroglandular intensity greater than twice fat intensity. The quantitative BPU tool was designed to provide values that reflect these definitions, such that it measures the ratio of average image counts (counts per pixel) in fibroglandular tissue versus fat.

As MBI examinations create functional images of radiotracer localization, they do not provide distinct anatomic landmarks of the breast from which to distinguish fibroglandular tissue and fat. However, as MBI is acquired in positions analogous to mammography (CC and MLO), a mammogram viewed in conjunction with MBI may be used to generally determine fibroglandular and fat locations. In this first approach to develop a quantitative tool for BPU, we used a corresponding mammogram to identify fibroglandular and fat regions, applied these regions to the MBI images, and determined the fibroglandular-to-fat count ratio on MBI as a quantitative BPU value, described in more detail as follows.

**Fig. 3:**
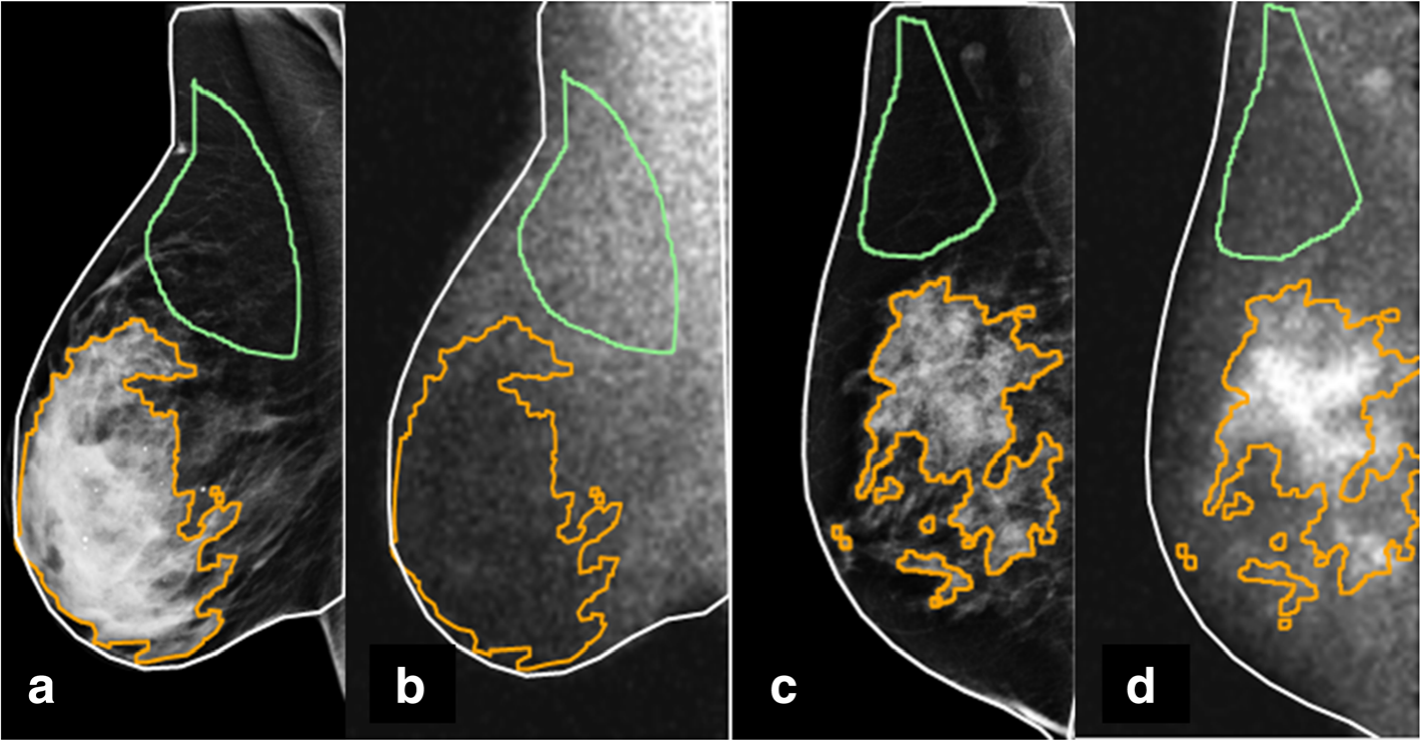
Example ROIs for quantitative BPU assessment. Breast images acquired in the mediolateral oblique position for two patients. Fibroglandular tissue is defined by the orange ROI and fat is defined by the green ROI. In panels **a** and **b**, the mammogram (**a**) and MBI (**b**) are shown for a patient classified as having photopenic BPU, measured quantitatively as 0.4. In panels **c** and **d**, the mammogram (**c**) and MBI (**d**) are shown for a patient with marked BPU, measured quantitatively as 2.4

An in-house software program was created to allow simultaneous display of MBI examinations with mammograms, as shown in Fig. 2. Using this program, each mammogram and its corresponding MBI images were processed as follows. First, a region of interest (ROI) outlining the entire breast visible on the mammogram was automatically drawn, based on a manually adjustable intensity threshold. Next, two other ROIs were drawn by the operator on the mammogram view—one to encompass an area predominantly made of fat and one to include predominantly fibroglandular tissue. The operator then manually adjusted the fibroglandular ROI using an intensity threshold to reject less-dense tissue, thereby reducing the overall size of the ROI and making it more specific to dense tissue. Finally, the three mammogram ROIs were copied to the corresponding MBI views and manually scaled and rotated as needed (all three as a single object) to register the outer outline of the mammogram to the MBI breast outline. This process was done to account for differences in breast position and compression force between the mammogram and MBI images. Example ROIs are shown in Fig. 3. The ratio of average counts (counts per pixel) in the fibroglandular ROI versus fat ROI was taken as a measure of quantitative BPU.

Two operators used this program to perform quantitative BPU measurements on each of the eight MBI views for all subjects. Each operator independently performed each step in the ROI process as described above, while blinded to the other operator’s results and blinded to patient identity and case status. One operator was a novice to medical imaging and image processing tools and the other operator was a nuclear medicine technologist familiar with mammography, MBI, and image processing software. To determine intraoperator agreement, one operator performed quantitative BPU measurements a second time on a sample of 48 subjects.

### Statistical analysis

Case and control characteristics are summarized using frequencies for categorical variables or mean and standard deviation (SD) with range for continuous variables. Conditional logistic regression was used to evaluate the primary hypothesis that quantitative BPU is associated with breast cancer. Models were adjusted for BMI and PD and postmenopausal hormone use. All ORs reported are for a one-unit change in quantitative BPU measurement. Analyses were repeated within premenopausal and postmenopausal subgroups.

Agreement in quantitative BPU across eight MBI views was assessed using several methods including intraclass correlation (ICC), principal component (PC) analysis, and a nested random effects model to inform the summary quantitative measure. ICC for each of the eight views was calculated and ranged from 0.74 to 0.92. PC analysis revealed a lack of multidimensionality among the eight views, as the first and second PCs explained 0.83 and 0.09 of the variation in all eight measures. Lastly, a nested random effects model demonstrated that only 2% of the variation in quantitative BPU was due to the eight views within a subject and 83% was intersubject variation, leaving 15% of variation due to random error. Therefore, with low dimensionality and limited variation across the eight images, we used an average of the quantitative BPU values from the eight views as the quantitative BPU measure for each subject herein. Analyses of individual views and other combinations (averages by breast side and view) along with their *p* values and area under the curves (AUCs) are summarized by operator in Additional file 1 (Table S1).

Interoperator and intraoperator agreement in quantitative BPU measures were summarized by ICC. Agreement between quantitative BPU and subjective BPU categories and PD measured on mammography was determined by Spearman and Pearson correlations, respectively. For all comparisons, *p* < 0.05 was considered statistically significant. Analyses were performed within SAS (Cary, NC; version 9.4).

## Results

### Subject characteristics

A total of 239 subjects, including 62 cases and 177 controls, were included in the study, with their characteristics presented in Table 1. Cases and controls were similar on matched variables of age and menopausal status, as expected. Cases and controls were not significantly different in any other characteristic examined, including BI-RADs density and PD. Quantitative BPU measures ranged from 0.41 to 3.18, with mean (IQR) values of 1.36 (1.09–1.54) for cases versus 1.18 (0.97–1.31) for controls for operator 1 (*p* = 0.002) and 1.36 (1.04–1.56) for cases and 1.19 (0.93–1.33) for controls for operator 2 (*p* = 0.007) (Table 2).

**Table 1:** Characteristics of breast cancer cases and controls, matched on age and menopausal status

Characteristic	Breast cancer cases (*n* = 62)	Controls (*n* = 177)	*P*
Age at MBI (years)^a^	60.3 ± 10.6 (38–88)	60.2 ± 10.4 (38–86)	NA
Menopausal status			NA
Premenopausal	13 (21)	38 (22)	
Postmenopausal	49 (79)	138 (78)	
Body mass index (kg/m^2^)^a^	27.7 ± 6.4 (18.8–55.5)	26.2 ± 4.7 (18.6–44.3)	0.06
Postmenopausal systemic hormone therapy^b^			0.57
Current use at MBI	13 (27)	44 (31)	
No current use at MBI	36 (73)	97 (69)	
BI-RADS density			0.81
Almost entirely fat	1 (2)	3 (2)	
Scattered fibroglandular densities	10 (16)	34 (19)	
Heterogeneously dense	44 (71)	113 (64)	
Extremely dense	7 (11)	26 (15)	
Percent density^a^	24.8 ± 8.3 (3.5–48.0)	24.6 ± 10.2 (1.8–53.8)	0.93
Tumor invasiveness			
Invasive	45 (73)	NA	
DCIS	17 (27)	NA	
Gail model 5-year risk^a^	2.7 ± 1.5 (0.6–7.2)	2.4 ± 1.5 (0.5–9.5)	0.23
BCSC model 5-year risk^a^	2.6 ± 1.2 (0.7–5.4)	2.3 ± 1.5 (0.4–13.2)	0.29
Family history of breast cancer			0.45
One or more first-degree relatives	33 (53)	86 (48)	
No first-degree relatives	29 (47)	93 (52)	
Personal history of biopsy showing atypia or LCIS			0.07
Yes	6 (10)	6 (3)	
No	56 (90)	173 (97)	

Unless otherwise noted, data are number of patients and data in parentheses are percentages

^a^Data are mean ± standard deviation; data in parentheses are the range

^b^Data are among postmenopausal women only (49 breast cancer cases; 138 controls)

*BCSC*, Breast Cancer Surveillance Consortium; *BI-RADS*, Breast Imaging-Reporting and Data System; *DCIS*, ductal carcinoma in situ; *LCIS*, lobular carcinoma in situ; *MBI*, molecular breast imaging; *NA*, not available

**Table 2:** Molecular breast imaging (MBI) quantitative background parencymal uptake (BPU) components by case status

Characteristic		Breast cancer cases	Controls	
	Operator	*n* = 62	*n* = 176	*P*
Average counts in fibroglandular tissue, mean ± SD (range)	1	41.5 ± 20.7 (6.8–116.6)	37.6 ± 23.2 (7.3–218.4)	0.08
	2	41.2 ± 21.1 (7.2–194.9)	36.5 ± 21.7 (6.5–114.6)	0.08
Average counts in fat, mean ± SD (range)	1	31.5 ± 14.3 (5.9–87.4)	32.7 ± 17.2 (6.2–95.7)	0.99
	2	32.6 ± 16.0 (5.7–90.7)	32.5 ± 17.0 (7.2–98.9)	0.78
Quantitative BPU (fibroglandular/fat), mean ± SD (range)	1	1.4 ± 0.4 (0.8, 2.8)	1.2 ± 0.3 (0.4, 2.9)	0.002
	2	1.4 ± 0.5 (0.8, 2.9)	1.2 ± 0.4 (0.5, 3.2)	0.007

### BPU agreement

The two operators showed good agreement in assessing quantitative BPU; the interoperator ICC for the average of the eight views was 0.92 (95% confidence interval (CI) 0.90–0.94) and ICCs ranged from 0.75 (95% CI 0.68–0.80) to 0.92 (95% CI 0.86–0.95) across the eight views. Intraoperator agreement in quantitative BPU was 0.98 (0.96–0.99) for the average of the eight views and ICCs ranged from 0.80 (95% CI 0.67–0.88) to 0.91 (95% CI 0.84–0.95) across the eight views.

**Fig. 4:**
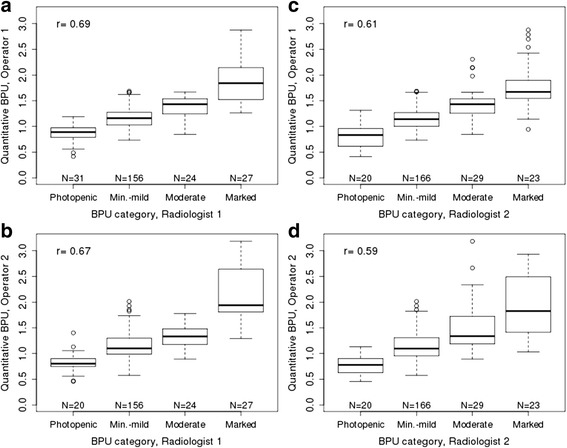
Quantitative background parencymal uptake (BPU) measurements by subjective BPU category. BPU as assessed by **a**) operator 1 versus radiologist reader 1, **b**) operator 2 versus radiologist reader 1, **c**) operator 1 versus radiologist reader 2, and **d**) operator 2 versus radiologist reader 2 with corresponding Spearman correlations (all *p* values< 0.0001)

Quantitative BPU measurements correlated well with subjective BPU categories previously assessed by radiologists [10], as shown in Fig. 4. Spearman correlations ranged from 0.59 to 0.69 (all *p* values < 0.0001) for the four combinations of two categorical BPU readers and two quantitative BPU operators.

**Fig. 5:**
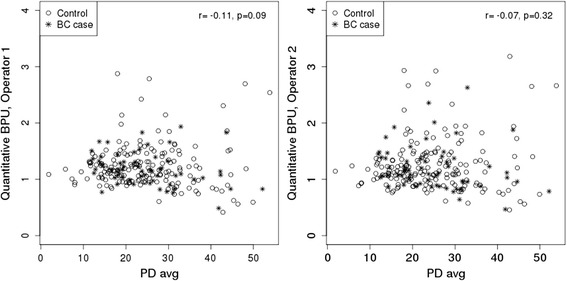
Quantitative background parencymal uptake (BPU) measurements by mammographic percent density (PD). BPU for breast cancer (BC) cases and controls are shown for both operators, with corresponding Spearman correlations

Quantitative BPU measures and PD showed weak or no correlation (all *p* values > 0.05 unless specified) in the total sample (operator 1: *r* = −0.11, operator 2: *r* = −0.07) (Fig. 5), or separately in cases (operator 1: *r* = 0.12, operator 2: *r* = 0.14), or controls (operator 1: *r* = −0.20, *p* = 0.009; operator 2: *r* = −0.13).

### Breast cancer cases versus controls

Quantitative BPU was associated with breast cancer risk in models adjusted for BMI, with odds ratios per 1 unit BPU of 3.70 (95% CI 1.54–8.92) and 2.37 (95% CI 1.19–4.70) for the two operators, respectively (Table 3). Results were similar for models including PD or postmenopausal hormone use.

**Table 3:** Association of quantitative background parencymal uptake (BPU) (per 1 unit BPU) with breast cancer

	OR (95% CI), adjusted for BMI	OR (95% CI), adjusted for BMI and PD	OR (95% CI), adjusted for BMI and postmenopausal hormones
Overall			
Operator 1	3.70 (1.54–8.92)	3.98 (1.58–10.05)	3.85 (1.58–9.38)
*p* value	0.0036	0.0034	0.0030
AUC (95% CI)	0.63 (0.56–0.71)	0.66 (0.59–0.73)	0.63 (0.56–0.70)
Operator 2	2.37 (1.19–4.70)	2.35 (1.17–4.71)	2.31 (1.17–4.55)
*p* value	0.0136	0.0163	0.0154
AUC (95% CI)	0.58 (0.51–0.66)	0.61 (0.54–0.69)	0.61 (0.54–0.69)
Postmenopausal women (*n* = 187: 49 cases, 138 controls)			
Operator 1	5.57 (1.62–19.08)	8.39 (2.10–33.55)	5.87 (1.69–20.36)
*p* value	0.0063	0.0026	0.0053
AUC (95% CI)	0.65 (0.57–0.73)	0.70 (0.62–0.78)	0.65 (0.57–0.73)
Operator 2	2.91 (1.15–7.35)	3.62 (1.34–9.79)	2.99 (1.18–7.57)
*p* value	0.0239	0.0113	0.0212
AUC (95% CI)	0.57 (0.48–0.65)	0.66 (0.58–0.74)	0.61 (0.53–0.69)
Premenopausal women (*n* = 51: 13 cases, 38 controls)			
Operator 1	2.42 (0.73–8.04)	2.09 (0.64–6.79)	NA
*p* value	0.1492	0.2224	
AUC (95% CI)	0.57 (0.41–0.73)	0.58 (0.42–0.73)	
Operator 2	1.70 (0.63–4.58)	1.47 (0.54–3.98)	NA
*p* value	0.2922	0.4468	
AUC (95% CI)	0.62 (0.47–0.78)	0.61 (0.45–0.76)	

*AUC*, area under the curve; *BMI*, body mass index; *CI*, confidence interval; *NA*, not applicable; *OR*, odds ratio; *PD*, percent density by Cumulus software

In analyses limited to postmenopausal women, quantitative BPU remained associated with breast cancer risk (BMI-adjusted OR per 1 unit BPU = 5.57, 95% CI 1.62–19.08, for operator 1, and 2.91, 95% CI 1.15–7.35, for operator 2); however, the BPU measure was not a statistically significant predictor for breast cancer in the premenopausal subset for either operator in a small sample of 13 cases.

Analysis of the eight MBI views separately by operator, as well as averages of right breast, left breast, MLO views, CC views, upper detector views, and lower detector views were considered (Additional file 1: Table S1). All models concluded that higher quantitative BPU was significantly associated with breast cancer risk with the exception of one view under operator 2 (OR = 1.5, *p* = 0.18). ORs ranged 2.0 to 4.5 for operator 1 and from 1.5 to 2.6 for operator 2, but reliably showed consistent overall model performance with AUCs from 0.57 to 0.62.

## Discussion

In this first evaluation of a simple region-of-interest tool for obtaining quantitative measurements of BPU on MBI, we found an association of quantitative BPU measurements with breast cancer risk, similar to that observed in a prior analysis of subjective BPU categories. This association was independent of mammographic density. In fact, in line with our previous observation that BPU can vary widely among women with similar mammographic density [10, 13], we saw no association between quantitative BPU and quantitative percent density in the current analysis.

The lack of relationship between BPU and mammographic density is not unexpected since BPU and density are fundamentally different imaging features. While density measures describe the amount of fibroglandular tissue in the breast by its anatomic appearance, BPU describes the functional radiotracer uptake within that fibroglandular tissue relative to the uptake in fat. Furthermore, density assessment tools, such as Cumulus, use a binary decision to categorize image pixels of a mammogram as “dense” or “non-dense”, and output the proportion of dense pixels, but do not take into account the intensity of those dense pixels. In contrast, BPU as measured on MBI is determined by the average intensity of the pixels in fibroglandular tissue relative to the intensity of pixels in fat. Therefore, it is possible for a breast to have a small amount of dense tissue and yet have high uptake within that dense tissue on MBI, resulting in high BPU. It is also possible for a breast to be very dense and have low BPU. The quantitative BPU value can vary substantially, even when percent density is similar. For instance, as seen in Fig. 5, women with percent density of about 40% were found to vary in quantitative BPU values from 0.4 to 3.2.

The underlying etiology relating BPU of Tc-99m sestamibi and risk of breast cancer is not yet known. In fact, the mechanism of Tc-99m sestamibi uptake in the breast in general is not well understood. Tc-99m sestamibi was developed as a tracer for imaging myocardial perfusion and was only incidentally discovered to accumulate in breast lesions in women undergoing cardiac testing [17]. Tc-99m sestamibi is known to be mostly sequestered in cellular mitochondria [8]. In breast cancer, its uptake is thought to reflect both blood flow to the tumor and mitochondrial status, which is affected by the cellular proliferation rate and apoptotic index [8, 9]. Benign breast lesions that are highly proliferative, such as atypical lesions and fibroadenomas, can also demonstrate high uptake of Tc-99m sestamibi that mimics breast cancer [18]. Although the etiology of variations in BPU among women has not been established, it can be hypothesized that breast fibroglandular tissue with higher blood flow and more proliferative cells would also exhibit higher BPU, and thus may represent tissue that is primed for breast cancer development.

Hormonal factors which are known to impact tissue perfusion and proliferation have been found to impact BPU. We have previously shown that high (moderate or marked) BPU is more prevalent among premenopausal women compared with postmenopausal women [13]. In postmenopausal women, those using exogenous hormonal therapy are more likely to have high BPU [13]. In the current study, quantitative BPU was strongly associated with breast cancer in postmenopausal women, but this association was somewhat attenuated with adjustment for hormone therapy use. In premenopausal women, BPU can fluctuate with the menstrual cycle, with higher levels of BPU observed in the luteal phase compared with the follicular phase [19]. When we restricted analysis to postmenopausal women in the current work, the association of quantitative BPU with breast cancer remained, suggesting that the association is not merely reflecting changes in BPU with the menstrual cycle. We did not observe a significant association in premenopausal women; however, the analysis was limited in power due to smaller numbers (*n* = 13 cases).

This study found that quantitative BPU assessed by operators correlates well with subjective BPU categories assessed by expert radiologists. We also found good agreement in quantitative BPU measurements between the two operators, one of whom was a novice to medical imaging, indicating that the quantitative method is robust and generalizable to other operators. Importantly, our results on the evaluation of eight views showed that the quantitative BPU obtained from any of the eight MBI views or any of the reported averages of multiple views is a reliable predictor of increased breast cancer risk, shown under two different operators with varying experience. Thus, future investigations could use a single view or combination of views for quantitative BPU assessment.

MBI is indicated for women with dense breasts, as reflected in our study population here where a majority of cases (82%) and controls (79%) were considered mammographically dense. In our institution’s practice, MBI is primarily used as a screening tool and is offered to women with dense breasts who seek supplemental screening but either do not wish to undergo or do not meet the high-risk criteria (20% lifetime risk by familial models) for screening breast magnetic resonance (MR) imaging. Supplemental screening MBI, performed with reduced administered doses of 300 MBq (8 mCi) Tc-99m sestamibi, offers a reported incremental cancer detection rate of 7.7 to 8.8 cancers per 1000 women screened [20, 21]. Although breast density is well-established as a breast cancer risk factor, for women with dense breasts included in this study there was no association between breast cancer and mammographic density assessed by mammographic categories or quantitative percent density. Thus, BPU will be an important risk factor for the dense breast population and may offer additional image-based risk information beyond density alone. Further work is needed to determine the impact of incorporating BPU into existing risk models.

Although our quantitative BPU method is relatively simple and easy to implement with minimal operator training, the method does have some limitations. First, given this was the first study relating the quantitative BPU to breast cancer, our estimates for the strength of the association were imprecise. This can be evidenced by the differential estimates of risk between operators (e.g., OR = 5.87 versus 2.99). These confidence intervals for the estimates are wide and overlapping. Further work is needed to develop a comprehensive model on a larger set of patients to ensure the risk estimates are properly calibrated.

Second, our method for measuring quantitative BPU currently requires user interaction to manually segment and align regions from the mammogram to the MBI. Best results are expected when the breast is similarly positioned on the mammogram and MBI, which is not always possible as they are acquired under separate examinations with the MBI performed under substantially less breast compression. Also, our current quantitative measure is based on the ratio of average pixel intensities in fibroglandular and fat regions obtained in two-dimensional planar images. Similar to findings from studies of mammographic density and breast cancer risk, a more precise or more reproducible risk association may be obtained if the BPU area is considered or volumetric BPU estimates are made. Future iterations of this method are anticipated to be automated and to evaluate additional factors such as BPU volume.

## Conclusions

Quantitative measurement of BPU, which can be reliably assessed by nonradiologist operators with a simple region-of-interest analysis tool, correlates well with subjective BPU categories assessed by expert radiologists. Similar to findings with subjective BPU categories, quantitative BPU measurement is associated with breast cancer risk, independent of mammographic density and hormonal factors. These results suggest that quantitative measures of BPU could serve as an additional tool for identifying a subset of women with mammographically dense breasts who are at greatest risk of breast cancer.

## Additional file

Additional file 1: **Table S1.** Association of quantitative BPU with breast cancer for each MBI view and combinations of views. (DOCX 18 kb)

## Abbreviations

ACR: American College of Radiology
AUC: Area under the curve
BI-RADS: Breast Imaging-Reporting and Data System
BMI: Body mass index
BPU: Background parenchymal uptake
CC: Craniocaudal
CI: Confidence interval
DCIS: Ductal carcinoma in situ
ICC: Intraclass correlation
IQR: Interquartile range
MBI: Molecular breast imaging
MLO: Mediolateral oblique
OR: Odds ratio
PC: Principal component
PD: Percent density
ROI: Region of interest
