# Supplementary material for: Quantitative background parenchymal uptake on molecular breast imaging and breast cancer risk: a case-control study
Source: Breast Cancer Res. 2018 Jun 5;20:46. doi: 10.1186/s13058-018-0973-3 (PMC5989426; doi:10.1186/s13058-018-0973-3)
Supplement: Supplementary file 1 — Table S1. Association of quantitative BPU with breast cancer for each MBI view and combinations of views. (DOCX 18 kb) [file 13058_2018_973_MOESM1_ESM.docx]

Table S1. Association of quantitative BPU with breast cancer for each MBI view and combinations of views.

|  |  |  |  |  |  |  |
| --- | --- | --- | --- | --- | --- | --- |
| Operator | Measure/view | Quantitative BPU in Controls  Mean (sd)  N=177 | Quantitative BPU in Cases  Mean (sd)  N=62 | OR (95% CI)* | p-value | AUC (95% CI) |
| Operator1 | Lower left MLO | 1.15 (0.44) | 1.28 (0.42) | 2.03 (1.00, 4.13) | 0.05 | 0.57 (0.50, 0.65) |
|  | Lower right MLO | 1.19 (0.39) | 1.41 (0.52) | 3.27 (1.57, 6.80) | 0.002 | 0.63 (0.56, 0.70) |
|  | Lower left CC | 1.15 (0.34) | 1.33 (0.47) | 2.90 (1.32, 6.37) | 0.008 | 0.64 (0.57, 0.71) |
|  | Lower right CC | 1.17 (0.34) | 1.37 (0.45) | 4.45 (1.75, 11.34) | 0.002 | 0.67 (0.60, 0.74) |
|  | Upper left MLO | 1.20 (0.41) | 1.34 (0.37) | 2.31 (1.08, 4.94) | 0.03 | 0.59 (0.52, 0.67) |
|  | Upper right MLO | 1.28 (0.40) | 1.49 (0.55) | 2.80 (1.43, 5.50) | 0.003 | 0.61 (0.54, 0.68) |
|  | Upper left CC | 1.13 (0.33) | 1.29 (0.37) | 3.16 (1.35, 7.39) | 0.008 | 0.62 (0.55, 0.70) |
|  | Upper right CC | 1.18 (0.36) | 1.35 (0.39) | 3.58 (1.51, 8.46) | 0.004 | 0.62 (0.55, 0.70) |
|  | 8 view avg | 1.18 (0.34) | 1.36 (0.41) | 3.70 (1.54, 8.92) | 0.004 | 0.63 (0.56, 0.71) |
|  | MLO views | 1.21 (0.39) | 1.38 (0.45) | 2.92 (1.35, 6.31) | 0.006 | 0.61 (0.54, 0.68) |
|  | CC views | 1.15 (0.33) | 1.33 (0.40) | 3.84 (1.56, 9.44) | 0.003 | 0.63 (0.56, 0.71) |
|  | Upper detector | 1.16 (0.34) | 1.35 (0.43) | 3.58 (1.51, 8.52) | 0.004 | 0.61 (0.53, 0.68) |
|  | Lower detector | 1.20 (0.34) | 1.37 (0.38) | 3.69 (1.54, 8.86) | 0.004 | 0.63 (0.56, 0.71) |
|  | Right breast | 1.20 (0.34) | 1.41 (0.45) | 4.10 (1.74, 9.68) | 0.001 | 0.63 (0.56, 0.71) |
|  | Left breast | 1.16 (0.35) | 1.31 (0.39) | 2.89 (1.25, 6.68) | 0.01 | 0.60 (0.52, 0.67) |
| Operator2 | Lower left MLO | 1.22 (0.47) | 1.38 (0.49) | 2.04 (1.07, 3.87) | 0.03 | 0.59 (0.52, 0.66) |
|  | Lower right MLO | 1.25 (0.54) | 1.48 (0.69) | 2.06 (1.22, 3.50) | 0.007 | 0.62 (0.54, 0.69) |
|  | Lower left CC | 1.11 (0.43) | 1.24 (0.46) | 1.83 (0.97, 3.50) | 0.07 | 0.60 (0.53, 0.67) |
|  | Lower right CC | 1.12 (0.46) | 1.34 (0.55) | 2.39 (1.28, 4.45) | 0.006 | 0.64 (0.57, 0.71) |
|  | Upper left MLO | 1.28 (0.47) | 1.40 (0.44) | 1.76 (0.92, 3.35) | 0.09 | 0.58 (0.50, 0.65) |
|  | Upper right MLO | 1.34 (0.55) | 1.54 (0.67) | 1.85 (1.12, 3.06) | 0.02 | 0.60 (0.53, 0.67) |
|  | Upper left CC | 1.09 (0.46) | 1.20 (0.44) | 1.51 (0.83, 2.77) | 0.18 | 0.60 (0.53, 0.67) |
|  | Upper right CC | 1.13 (0.52) | 1.30 (0.49) | 1.83 (1.05, 3.19) | 0.03 | 0.62 (0.55, 0.69) |
|  | 8 view avg | 1.19 (0.42) | 1.36 (0.46) | 2.37 (1.19, 4.70) | 0.01 | 0.58 (0.51, 0.66) |
|  | MLO views | 1.27 (0.48) | 1.45 (0.54) | 2.14 (1.16, 3.96) | 0.02 | 0.63 (0.56, 0.70) |
|  | CC views | 1.11 (0.44) | 1.27 (0.46) | 2.03 (1.08, 3.84) | 0.03 | 0.62 (0.55, 0.69) |
|  | Upper detector | 1.21 (0.43) | 1.36 (0.44) | 2.11 (1.08, 4.13) | 0.03 | 0.59 (0.52, 0.66) |
|  | Lower detector | 1.18 (0.43) | 1.36 (0.49) | 2.59 (1.29, 5.18) | 0.007 | 0.62 (0.55, 0.69) |
|  | Right breast | 1.22 (0.47) | 1.42 (0.54) | 2.12 (1.09, 4.13) | 0.03 | 0.51 (0.51, 0.66) |
|  | Left breast | 1.18 (0.41) | 1.31 (0.42) | 2.39 (1.23, 4.65) | 0.01 | 0.59 (0.52, 0.66) |

*Odds ratios are adjusted for body mass index.
